# Correction for Parra et al., “Isolation and characterization of novel plasmid-dependent phages infecting bacteria carrying diverse conjugative plasmids”

**DOI:** 10.1128/spectrum.00522-25

**Published:** 2025-08-20

**Authors:** Boris Parra, Bastiaan Cockx, Veronika T. Lutz, Lone Brøndsted, Barth F. Smets, Arnaud Dechesne

## AUTHOR CORRECTION

Volume 12, no. 1, e02537-23, 2024, https://doi.org/10.1128/spectrum.02537-23. 

Page 4: Fig. 2A of the original manuscript presented intergenomic similarities between our phages Hi226 and Lu221 and related phages that did not factor in the coverage, and which, therefore, cannot be used to reliably delineate taxa. This analysis redone using VIRIDIC (Moraru et al., 2020) yielded significantly lower value of intergenomic similarities (see Fig. 1 below). These corrected data indicate that, contrary to what was indicated in the *Results and Discussion* section of the original manuscript, phages Lu221 and Hi226 do not belong to the same genus as pSal-SNUABM-01, 7-11, and SE131.

**Fig 1:**
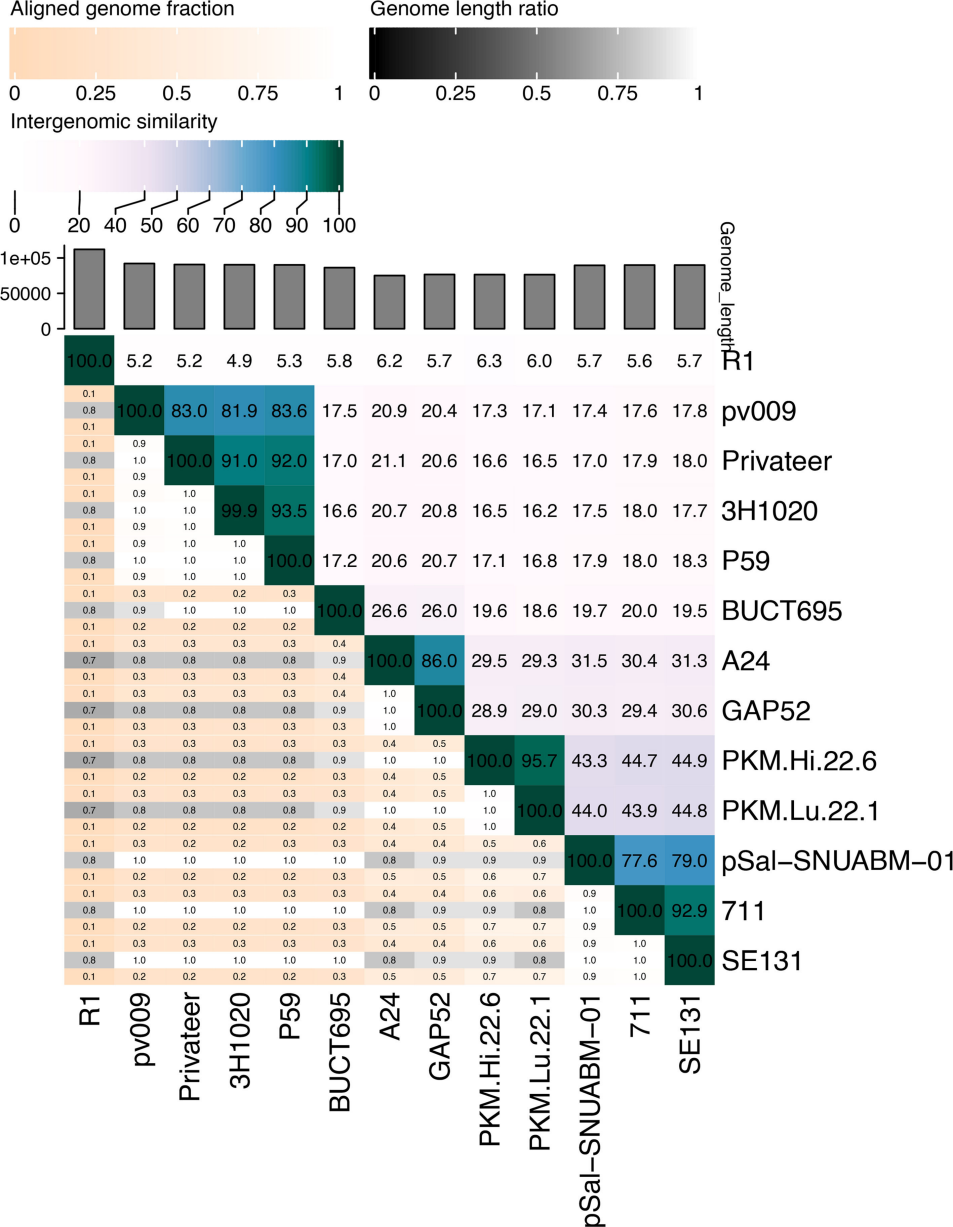
Heatmap of pairwise intergenomic similarities of phages Lu221, Hi226, and other members of the family *Grimontviridae*.

Page 5, paragraph 2: line 5 should read “According to our results, phages Lu221 and Hi226 form a novel genus, most closely related to the recently proposed genus *Moazamivirus* with phages pSal-SNUABM-01, 7-11, and SE131 as members (36).”

