# Supplementary material for: Isolation and characterization of novel plasmid-dependent phages infecting bacteria carrying diverse conjugative plasmids
Source: Microbiol Spectr. 2023 Dec 8;12(1):e02537-23. doi: 10.1128/spectrum.02537-23 (PMC10782986; doi:10.1128/spectrum.02537-23)
Supplement: Supplemental material — Tables S1 to S3; Fig. S1. [file spectrum.02537-23-s0001.docx]

**Supplemental material**

Table S1. Bacterial strains and conjugative plasmids used in this study. Antibiotic resistance determinants of each conjugative plasmid are included.

| Bacterial strain | | |
| --- | --- | --- |
| *Salmonella enterica* MHM112 | | |
| *Escherichia coli* K12 | | |
| *Kluyvera* sp. | | |
| *Enterobacter* sp. | | |
| *Pseudomonas putida* KT2440 | | |
| Plasmid | Antibiotic resistance | Reference |
| pKM101 | Ampicillin and rifampicin | 1 |
| RP4 | Tetracycline, ampicillin and kanamycin | 2 |
| drR27 | Nalidixic acid and tetracycline | 3 |
| pKJK5 | Tetracycline and trimethoprim | 4 |
| pIE321 | Tetracycline and streptomycin | 5 |
| R1drd19 | Ampicillin, chloramphenicol, kanamycin, streptomycin and sulphonamides | 6 |
| R477-1 | Streptomycin, tetracycline, chloramphenicol, kanamycin and sulphonamides | 7 |
| pOXA436 | Temocillin, piperacillin, ceftazidime, aztreonam, ertapenem and sulphonamides | 8 |
| R64 | Streptomycin and tetracycline | 9 |
| R388 | Trimethoprim and sulfonamide | 10 |
| R721 | Trimethoprim and streptomycin | 11 |
| pTR4 | Ampicillin, piperacillin, cefazolin, cefpodoxime, imipenem and meropenem | 12 |
| RN3 | Streptomycin and tetracycline | 13 |
| pB10 | Amoxicillin, streptomycin, sulfonamides and tetracycline | 14 |
| R6K | Ampicillin and streptomycin | 15 |

Plasmid pKM101 is a derivate of R46. Plasmid drR27 is a derepressed version of plasmid R27. Plasmid R1drd19 is a derepressed version of plasmid R1.

Table S2. Functional similarity of proteins encoded by phage Lu221 and pSal-SNUABM-01.

| Group | Function | Identity | start | stop | aa | strand |
| --- | --- | --- | --- | --- | --- | --- |
| Structural and packing related proteins | Terminase, large subunit | 91.70% | 7164 | 8648 | 494 | + |
|  | Portal protein | 79.37% | 8670 | 11033 | 787 | + |
|  | Scaffolding protein | 80.66% | 11264 | 12319 | 351 | + |
|  | Major capsid protein | 93.57% | 12445 | 13389 | 314 | + |
|  | Major capsid protein | 92.50% | 13466 | 15802 | 778 | + |
|  | Tail tubular protein | 87.34% | 16375 | 17064 | 229 | + |
|  | Tail fiber protein | 58.53% | 17074 | 17853 | 259 | + |
|  | Tail formation protein GpI | 74.43% | 17846 | 20734 | 962 | + |
|  | Structural protein | 72.97% | 21507 | 22376 | 289 | + |
|  | Tail fiber protein | 78.38% | 24096 | 24320 | 74 | + |
|  | DNA injection protein | 78.20% | 24333 | 25565 | 410 | + |
|  | Inner membrane subunit | 52.86% | 33381 | 33800 | 139 | + |
|  | Thioredoxin | 72.04% | 51587 | 51297 | 96 | - |
| Nucleotide metabolism related proteins | DNA stabilization protein | 77.66% | 22390 | 24096 | 568 | + |
|  | Endonuclease | 83.57% | 32959 | 33384 | 141 | + |
|  | DNA recombination-mediator protein | 80.70% | 36514 | 36170 | 114 | - |
|  | Acetyl transferase | 62.50% | 37137 | 36697 | 146 | - |
|  | DNA ligase | 66.57% | 38777 | 37686 | 363 | - |
|  | Exonuclease | 68.47% | 40112 | 39141 | 323 | - |
|  | RNA polymerase ECF-type sigma factor | 81.77% | 40861 | 40304 | 185 | - |
|  | Serine/threonine protein phosphatase | 77.92% | 43345 | 42818 | 175 | - |
|  | Endonuclease | - | 44179 | 43847 | 110 | - |
|  | DNA polymerase | 79.80% | 47182 | 45338 | 614 | - |
|  | Endonuclease | 74.39% | 48269 | 47913 | 118 | - |
|  | DPS protein (DNA binding protein) | 50.00% | 49350 | 48901 | 149 | - |
|  | Thymidylate synthase | 73.76% | 50006 | 49350 | 218 | - |
|  | Phosphate starvation-inducible protein PhoH, predicted ATPase | 72.57% | 52345 | 51587 | 252 | - |
|  | 2'-deoxycytidine 5'-triphosphate deaminase | 75.76% | 53412 | 52801 | 203 | - |
|  | DNA polymerase | 63.47% | 54093 | 53596 | 165 | - |
|  | DNA primase/helicase | 85.95% | 55899 | 54103 | 598 | - |
|  | Gamma-glutamyl cyclotransferase | 62.78% | 56632 | 55967 | 221 | - |
|  | Glutamine amidotransferase | 59.74% | 62219 | 60228 | 663 | - |
|  | Amidoligase | 62.66% | 63943 | 62276 | 555 | - |
|  | COOH.NH2 ligase-type 2 | 74.53% | 65324 | 64521 | 267 | - |
|  | Anti-RecBCD protein | 40.21% | 69658 | 69353 | 101 | - |
|  | ProQ/FINO family protein | 49.13% | 70681 | 69929 | 250 | - |
| Lysis | Endolysin (L-alanyl D-glutamate peptidase) | 73.08% | 42815 | 42411 | 134 | - |
|  | Endolysin | 67.14% | 68317 | 67865 | 150 | - |
| Additional function | ATP grasp protein | 46.77% | 59675 | 58488 | 395 | - |

**Table S3. Host range of phages Lu221 and Hi226.** Bacterial hosts are listed with the plasmids carried by them. Numbers correspond to the average of plaque-counting data from triplicate experiments, as plaque forming units PFU mL^-1^ with error range.

| Bacterial host | Plasmid | Inc | Hi226 (PFU mL^-1^) | Lu221 (PFU mL^-1^) |
| --- | --- | --- | --- | --- |
| *S. enterica* MHM112 | drR27 | H | 6.8x10^6^ ± 1.0x10^6^ | 5.7x10^6^ ± 4.7x10^5^ |
| *S. enterica* MHM112 | pKM101 | N | 6.5x10^7^ ± 3.6x10^7^ | 7.5x10^7^ ± 2.4x10^7^ |
| *S. enterica* MHM112 | pKJK5 | P | 1.0x10^7^ ± 6.2x10^5^ | 9.0x10^6^ ± 2.2x10^6^ |
| *S. enterica* MHM112 | RP4 | P | 1.7x10^7^ ± 2.3x10^6^ | 1.2x10^7^ ± 5.2x10^6^ |
| *S. enterica* MHM112 | R1drd19 | F | 6.7x10^6^ ± 9.4x10^5^ | 7.7x10^6^ ± 2.2x10^6^ |
| *E. coli* K12 | drR27 | H | 0 | 0 |
| *E. coli* K12 | R477-1 | H | 6.2x10^6^ ± 1.0x10^6^ | 5.0x10^6^ ± 7.1x105 |
| *E. coli* K12 | pOXA436 | H | 6.7x10^7^ ± 1.3x10^7^ | 6.5x10^7^ ± 2.3x10^7^ |
| *E. coli* K12 | R64 | I | 2.8x10^6^ ± 1.9x10^6^ | 4.0x10^6^ ± 2.1x10^6^ |
| *E. coli* K12 | R721 | I | 1.2x10^5^ ± 8.5x10^4^ | 1.3x10^5^ ± 4.7x10^4^ |
| *E. coli* K12 | pTR4 | N | 2.8x10^5^ ± 1.2x10^5^ | 4.7x10^5^ ± 1.5x10^5^ |
| *E. coli* K12 | RN3 | N | 4.8x10^5^ ± 2.5x10^5^ | 5.0x10^5^ ± 2.3x10^5^ |
| *E. coli* K12 | pKM101 | N | 8.3x10^6^ ± 4.4x10^6^ | 7.5x10^5^ ± 1.9x10^6^ |
| *E. coli* K12 | pB10 | P | 0 | 0 |
| *E. coli* K12 | RP4 | P | 0 | 0 |
| *E. coli* K12 | pKJK5 | P | 0 | 0 |
| *E. coli* K12 | R388 | W | 0 | 0 |
| *E. coli* K12 | pIE321 | W | 8.7x10^6^ ± 2.3x10^6^ | 6.8x10^6^ ± 2.6x10^6^ |
| *E. coli* K12 | R6K | X | 2.0x10^6^ ± 8.2x10^5^ | 2.0x10^6^ ± 8.2x10^5^ |
| *Kluyvera* | drR27 | H | 1.3x10^6^ ± 1.5x10^6^ | 1.3x10^6^ ± 1.5x10^6^ |
| *Kluyvera* | pKM101 | N | 0 | 0 |
| *Kluyvera* | pKJK5 | P | 3.3x10^6^ ± 1.2x10^6^ | 4.5x10^6^ ± 1.1x10^6^ |
| *Kluyvera* | R1drd19 | F | 4.7x10^6^ ± 1.0x10^6^ | 4.3x10^6^ ± 8.5x10^6^ |
| *Enterobacter* | pKM101 | N | 6.2x10^6^ ± 3.3x10^6^ | 5.8x10^6^ ± 1.2x10^6^ |
| *Enterobacter* | drR27 | H | 7.7x10^5^ ± 2.2x10^5^ | 7.8x10^5^ ± 1.6x10^5^ |
| *P. putida* KT2440 | RP4 | P | 0 | 0 |
| *P. putida* KT2440 | pKJK5 | P | 0 | 0 |
| *P. putida* KT2440 | R388 | W | 0 | 0 |

**Figure S1**. Example of small drop plaque assay system used for the host range determination. Phage Hi226 infecting *S. enterica* carrying the conjugative plasmid drR27 (A); phage Hi226 infecting *S. enterica* carrying the conjugative plasmid pKJK5 (B) and phage Hi226 infecting *S. enterica* carrying the conjugative plasmid R1drd19 (C). All assays were done by triplicate using four different phage concentrations, diluted in log_10_ (-1, -2, -3 and -4).


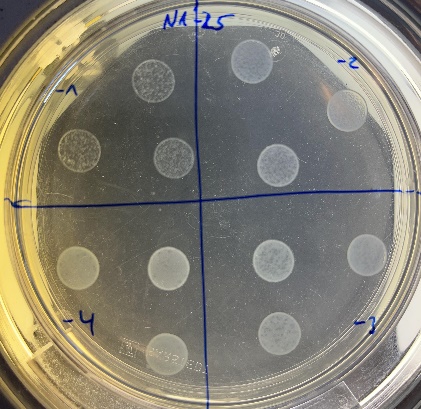


**A**


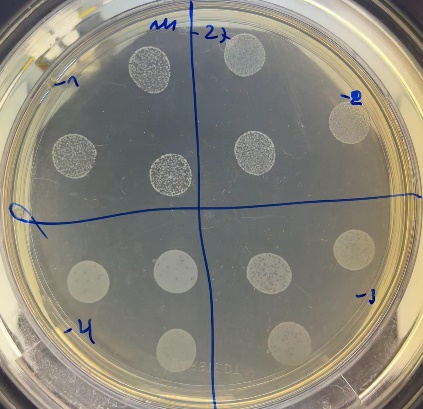


**C**


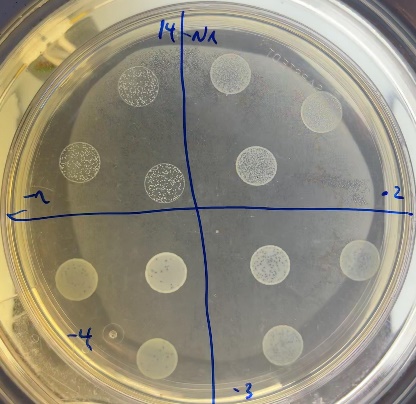


**B**

**References**

1. Yeo, H. J., Yuan, Q., Beck, M. R., Baron, C., & Waksman, G. (2003). Structural and functional characterization of the VirB5 protein from the type IV secretion system encoded by the conjugative plasmid pKM101. Proceedings of the National Academy of Sciences, 100(26), 15947-15952.
2. Wang, Q., Mao, D., & Luo, Y. (2015). Ionic liquid facilitates the conjugative transfer of antibiotic resistance genes mediated by plasmid RP4. Environmental Science & Technology, 49(14), 8731-8740.
3. Sherburne, C. K., Lawley, T. D., Gilmour, M. W., Blattner, F. R., Burland, V., Grotbeck, E., ... & Taylor, D. E. (2000). The complete DNA sequence and analysis of R27, a large IncHI plasmid from Salmonella typhi that is temperature sensitive for transfer. Nucleic acids research, 28(10), 2177-2186.
4. Bahl, M. I., Hansen, L. H., Goesmann, A., & Sørensen, S. J. (2007). The multiple antibiotic resistance IncP-1 plasmid pKJK5 isolated from a soil environment is phylogenetically divergent from members of the previously established α, β and δ sub-groups. Plasmid, 58(1), 31-43.
5. Revilla, C., Garcillán-Barcia, M. P., Fernández-López, R., Thomson, N. R., Sanders, M., Cheung, M., ... & de la Cruz, F. (2008). Different pathways to acquiring resistance genes illustrated by the recent evolution of IncW plasmids. Antimicrobial agents and chemotherapy, 52(4), 1472-1480.
6. Dionisio, F., Matic, I., Radman, M., Rodrigues, O. R., & Taddei, F. (2002). Plasmids spread very fast in heterogeneous bacterial communities. Genetics, 162(4), 1525-1532.
7. Gama, J. A., Zilhão, R., & Dionisio, F. (2017). Conjugation efficiency depends on intra and intercellular interactions between distinct plasmids: plasmids promote the immigration of other plasmids but repress co-colonizing plasmids. Plasmid, 93, 6-16.
8. Samuelsen, Ø., Hansen, F., Aasnæs, B., Hasman, H., Lund, B. A., Leiros, H. K. S., ... & Hammerum, A. M. (2018). Dissemination and characteristics of a novel plasmid-encoded carbapenem-hydrolyzing class D β-lactamase, OXA-436, found in isolates from four patients at six different hospitals in Denmark. Antimicrobial Agents and Chemotherapy, 62(1), 10-1128.
9. Kim, S. R., & Komano, T. (1997). The plasmid R64 thin pilus identified as a type IV pilus. Journal of bacteriology, 179(11), 3594-3603.
10. Llosa, M., Bolland, S., & de la Cruz, F. (1994). Genetic organization of the conjugal DNA processing region of the IncW plasmid R388. Journal of molecular biology, 235(2), 448-464.
11. Kim, S. R., & Komano, T. (1992). Nucleotide sequence of the R721 shufflon. Journal of bacteriology, 174(21), 7053-7058.
12. Chen, Y. T., Lin, A. C., Siu, L. K., & Koh, T. H. (2012). Sequence of closely related plasmids encoding bla NDM-1 in two unrelated Klebsiella pneumoniae isolates in Singapore. PLoS One, 7(11), e48737.
13. Datta, N., & Hedges, R. W. (1971). Compatibility groups among fi− R factors. Nature, 234(5326), 222-223.
14. Schluter, A., Heuer, H., Szczepanowski, R., Forney, L. J., Thomas, C. M., Puhler, A., & Top, E. M. (2003). The 64 508 bp IncP-1 β antibiotic multiresistance plasmid pB10 isolated from a waste-water treatment plant provides evidence for recombination between members of different branches of the IncP-1 β group. Microbiology, 149(11), 3139-3153.
15. Núñez, B., Avila, P., & De La Cruz, F. (1997). Genes involved in conjugative DNA processing of plasmid R6K. Molecular microbiology, 24(6), 1157-1168.
